# Recent advances in understanding non-celiac gluten sensitivity

**DOI:** 10.12688/f1000research.15849.1

**Published:** 2018-10-11

**Authors:** Maria Raffaella Barbaro, Cesare Cremon, Vincenzo Stanghellini, Giovanni Barbara

**Affiliations:** 1 Department of Medical and Surgical Sciences (DIMEC) and Center for Applied Biomedical Research (CRBA), Alma Mater Studiorum - University of Bologna and S. Orsola-Malpighi Hospital, Bologna, Italy

**Keywords:** NCGS, gluten, wheat, diagnosis, IBS

## Abstract

Non-celiac gluten sensitivity (NCGS) is a condition characterized by intestinal and extra-intestinal symptoms related to the ingestion of gluten-containing foods in the absence of celiac disease and wheat allergy. The diagnosis is cumbersome and currently confirmed only by gluten withdrawal and double-blind placebo challenge protocols. There is great overlap in symptoms between NCGS and other functional gastrointestinal disorders, making a differential diagnosis difficult. The pathophysiology of NCGS is largely unclear, and there are contrasting data on the trigger of this condition. This review will highlight the state-of-the-art knowledge on NCGS and the key open questions.

## Introduction

Non-celiac gluten sensitivity (NCGS) is a condition characterized by intestinal and extra-intestinal symptoms triggered by the introduction of gluten-containing foods ^1^. Despite the efforts that have been made, this condition remains ill defined. It is still a matter of debate whether NCGS is caused by gluten or other components of wheat. The prevalence rate of NCGS is unknown but is suspected to be higher than that of celiac disease. In the absence of a reliable biomarker, confirmation of an NCGS diagnosis can be made only with a double-blind placebo-controlled (DBPC) gluten challenge. However, this procedure is complex and of limited applicability in routine clinical practice. In the absence of diagnostic markers, distinguishing NCGS from functional gastrointestinal (GI) disease—principally, irritable bowel syndrome (IBS)—is a challenge. In this review, we discuss recent advances in the pathophysiology, epidemiology, diagnosis, and treatment of NCGS.

## Definition and epidemiology

NCGS is a condition characterized by intestinal and extra-intestinal symptoms related to the ingestion of gluten-containing foods in patients in whom celiac disease and wheat allergy have been excluded ^1^. Some authors indicated that the terminology “non-celiac wheat sensitivity” is more appropriate, as components other than gluten could be implicated in symptom experience. Although NCGS has been suggested to be the most common gluten-related disorder, its prevalence remains unknown because of a lack of diagnostic markers. According to self-reported data, the prevalence rate of NCGS ranges between 0.5% and 13% in the general population ^2– 5^, and prevalence is higher in women ^2, 3^, teenagers, and patients in the third to fourth decade of life ^2, 4^.

## Pathophysiology

The pathophysiology of NCGS is largely undetermined, as most studies were performed in patients in whom symptoms were self-reported. Under these circumstances, NCGS remains a subjective diagnosis influenced by nocebo response. With these limitations, some studies have investigated the implication of wheat components and possible mechanisms underlying bowel dysfunction and symptom generation. It has been suggested that gluten is not the only trigger of symptoms in NCGS. Other components include amylase-trypsin inhibitors (ATIs) and fermentable oligo-, di-, and mono-saccharides and polyols (FODMAPs).

### Gluten

Gluten proteins represent the major storage proteins of wheat, barley, and rye which are in the endosperm of the grains. Gluten proteins are prolamins and are rich in glutamine and proline and have different names according to grain species, including (a) gliadins (monomers) and glutenins (polymers) in wheat, (b) hordeins in barley, and (c) secalins in rye ^6^. After ingestion, gluten is hydrolyzed by GI proteases, but the abundance of glutamine and proline residues produces incomplete gluten digestion. Previous studies have shown that gliadin, even in healthy subjects, can determine a prompt and transient increase in gut permeability which is related to the amount of the peptide ingested with the diet ^7, 8^. The increase in intestinal permeability is thought to be the consequence of binding of gliadin to the CXCR3 chemokine receptor. This in turn determines the release of zonulin, a facilitator of interepithelial tight junction opening. These observations have been made in single centers and need to be confirmed. In turn, the passage of indigested peptides through the epithelium increases, resulting in over-stimulation of the immune system in the lamina propria. Glutamine and proline are the preferred substrate of mucosal tissue transglutaminases (tTGs). The result of tTG enzymatic breakdown generates peptides with a great affinity for major histocompatibility complex II (MHC II), which strongly stimulate the immune system in HLA-DQ2/8-positive subjects ^9^. In celiac disease, at least, the antigen presentation to T cells induces innate and adaptive responses that culminate in villus atrophy, crypt hyperplasia, and enhanced infiltration of intra-epithelial lymphocytes ^10, 11^.

The role of gluten as a trigger of NCGS symptoms is supported by different lines of evidence. Di Sabatino *et al*. performed a randomized, placebo-controlled, crossover trial in 59 patients with self-diagnosed gluten sensitivity and demonstrated that symptoms are worse in patients challenged with gluten (4.375 g) compared with those challenged with placebo ^12^. In a large trial including patients with functional GI symptoms, 14% of these were diagnosed with NCGS and had symptom worsening during a 5.6 g/day challenge of gluten ^13^. In a pediatric population of 1,114 children with functional GI symptoms negative for celiac disease and wheat allergy, 28 patients self-reporting NCGS performed a double-blind gluten (10 g/day) challenge and 11 patients (39%) were NCGS positive. These data showed that gluten challenge-confirmed NCGS is not rare in children who have functional bowel disorders ^14^. However, others have suggested that in case of a high nocebo effect, the methodology used to define NCGS should be chosen carefully ^15^. In a controlled study, only one-third of patients with self-reported NCGS had confirmation of the diagnosis with a double-blind gluten challenge, suggesting that most patients with self-reported diagnosis should not be labeled as NCGS. Interestingly, only one-third of these patients classified as NCGS could recognize the flour containing gluten ^16^. The effect of gluten and fructans on symptoms was evaluated in individuals with self-reported NCGS, and no effect of gluten emerged compared with placebo ^17^.

### FODMAPs

FODMAPs are part of wheat and may play a role in NCGS pathophysiology and symptom development. In a placebo-controlled crossover re-challenge study, it was found that GI symptoms improved during reduced FODMAP intake and worsened with gluten or whey protein. The effect of gluten on symptoms was observed in only 8% of subjects. These data suggest that most effects described as gluten related in patients with NCGS could in fact be determined by the presence of other components contained in wheat, including FODMAPs ^18^. A more recent article demonstrated that two weeks of a low-FODMAP diet in patients with self-reported NCGS significantly improved symptoms. In addition, this study showed that two weeks of a gluten-free diet (GFD) induced a symptom reduction compared with that reported during the low-FODMAP diet ^19^. Attention has been directed to fructans, a component of FODMAPs. In a controlled challenge with gluten, fructans, or placebo, it was shown that the highest symptom scores were recorded during fructan challenge but that no difference emerged between gluten and placebo ^17^.

### Amylase-trypsin inhibitors

Gluten represents 80% to 90% of the total amount of grain proteins. The remaining 10% to 20% is represented by albumins and globulins. ATIs, which represent up to 4% of total protein, are major allergens in baker’s asthma ^20^ and activators of the innate immune response *in vitro* and *in vivo* ^21^. ATIs induce intestinal infiltration and activation of myeloid cells and the release of inflammatory cytokines. Interestingly, the amount of ATIs is increased in modern hexaploid wheat, rye, and barley compared with older wheat variants ^22^. Zevallos *et al*. demonstrated that ATIs are highly resistant to proteases and heat, activate Toll-like receptor-4, and can evoke intestinal inflammation by activating gut and mesenteric lymph node myeloid cells ^22^. Based on this evidence, it has been suggested that ATIs may contribute to the activation of innate immune cells in low-level pre-existing small intestinal and colonic inflammation and could have a role in the pathophysiology of NCGS ^22^.

## Clinical presentation

In general, symptoms in patients with NCGS appear with the ingestion of gluten and disappear or ameliorate with gluten avoidance. The re-introduction of gluten with diet or gluten challenge determines symptom reappearance. The main symptoms in patients with NCGS include abdominal bloating and pain in the upper or lower abdomen, diarrhea, nausea, aphthous stomatitis, alternating bowel habits, and constipation. These symptoms are also common in other functional GI disorders, particularly IBS and functional dyspepsia. Differentiation of NCGS from functional symptoms represents a challenge for clinicians. For this reason, some authors argue that, in the absence of a reliable biomarker, NCGS may not exist as a distinct clinical entity and that symptom experience is the result of symptoms worsening with diet, as often occurs in IBS or dyspepsia. In addition to experiencing GI symptoms, patients with NCGS most often experience a complex of extra-intestinal symptoms, including a “foggy mind”, which is described as an inability to concentrate, reduction of mnemonic capabilities, and lack of well-being as well as tiredness, headache, anxiety, numbness, joint/muscle pain, and skin rash/dermatitis ^23^.

## Diagnosis

The diagnosis of NCGS remains elusive because of poor knowledge of the mechanisms underlying symptom experience and the lack of reliable biomarkers. According to the Salerno consensus conference, the gold standard for the diagnosis of NCGS is based on a DBPC crossover gluten challenge ^1^. This diagnostic process is complex and remains unfeasible in daily clinical practice.

It is still unclear whether patients with a diagnosis of NCGS should follow a GFD for life. This is particularly relevant in light of the fact that intestinal and extra-intestinal symptoms may persist in around 70% of patients after one year of GFD ^24^. It is still unclear whether gluten reduction, rather than avoidance, would be enough to control symptoms. Some authors have criticized the efficacy of DBPC in identifying patients with NCGS. Molina-Infante and Carroccio analyzed data from 10 DBPC gluten challenge trials comprising 1,312 adults and demonstrated that only 16% of patients showed gluten-specific symptoms and 40% of these subjects had a nocebo response. This evidence reveals heterogeneity and potential methodology flaws among studies of gluten challenge ^5^.

## Differential diagnosis

The differential diagnosis for NCGS should focus on celiac disease, wheat allergy, and functional GI syndromes such as IBS. The diagnoses of celiac disease and wheat allergy are discussed in detail in previous comprehensive reviews and will not be discussed further ^25, 26^.

**Figure 1.**
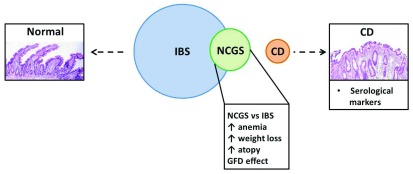
Overlap and differences between irritable bowel syndrome (IBS), non-celiac gluten sensitivity (NCGS), and celiac disease (CD). There is an overlap in symptoms between NCGS and IBS patients, although there are also some differences. NCGS symptoms improve after a gluten-free diet (GFD). In addition, NCGS is associated with a greater incidence of anemia, and weight loss and atopy are more common in patients with NCGS than in patients with IBS. NCGS could be considered a partially separated entity from IBS but is completely distinct from CD for serological and histological manifestations.

Symptoms of NCGS overlap with those of IBS. In the absence of valid biomarkers, differential diagnosis between the two conditions is not always feasible in daily clinical practice ( Figure 1). Gluten, the key determinant factor in NCGS, is sometimes identified by patients with IBS as a major trigger of symptoms ^27, 28^. In a UK study evaluating the prevalence of self-reported NCGS, it was found that individuals with gluten sensitivity fulfilled the Rome III criteria for IBS to a greater extent than those without gluten sensitivity (20% versus 4%) ^29^. Carroccio *et al*. showed that one-third of patients with IBS undergoing a DBPC wheat challenge were sensitive to wheat ^23^. A randomized DBPC gluten re-challenge trial in patients with IBS demonstrated that gluten significantly worsened overall symptoms, abdominal pain, abdominal bloating, tiredness, and satisfaction with stool consistency. Symptom improvement was not associated with HLA genotype ^30^. Compared with IBS, NCGS is characterized by a greater incidence of anemia ^23^, weight loss ^23^, atopy ^31^, and anti-gliadin (AGA) IgG antibody ^32^. The HLA-DQ2/8 phenotype has been reported to be variable, from 24% to 100%, in patients with NCGS ^5^. Vazquez-Roque *et al*. performed a randomized controlled trial of a gluten-containing diet (GCD) or GFD in patients with IBS with diarrhea (IBS-D) and showed that GCD was associated with increased small bowel permeability and decreased tight junction expression in the colonic mucosa ^33^. In addition, the effect of gluten on epithelial permeability was greater in HLA-DQ2/8-positive patients ^33^. Using confocal laser endomicroscopy, Fritscher-Ravens *et al*. showed that wheat challenge in the duodenal mucosa of IBS-D patients with suspected wheat intolerance induced an increase in intra-epithelial lymphocytes, epithelial breaks, and inter-villous spaces ^34^. These data suggest that wheat can determine morphological changes in the intestinal wall of patients with self-reported wheat intolerance. A recent systematic review evaluated the efficacy of exclusion diet in IBS by the examination of randomized controlled trials. Nine studies turned out to be eligible. A GFD was evaluated in two studies and was associated with a reduction of global symptoms compared with a control diet (relative risk = 0.42), although the statistical significance was not reached. The remaining seven studies evaluated the efficacy of a low-FODMAP diet compared with different control interventions; only three of these studies used rigorous control diets and showed the least magnitude of effect. The authors concluded that based on the available data there is insufficient evidence to recommend a GFD or low-FODMAP diet in patients with IBS ^35^.

## Identification of putative biomarkers

Biomarkers are objectively measurable indicators of normal or pathological processes or pharmacological responses to a therapeutic intervention ^36^. The identification of an NCGS biomarker would represent an important advancement in the field of NCGS, as it would legitimize NCGS and advance diagnosis with the identification of relevant subgroups of patients responding to GFD.

For the time being, no valid biomarkers have been characterized, although some efforts have been made. Between 25% and 56% of subjects with self-reported NCGS had positive (>50 arbitrary units) AGA IgG antibodies ^32, 37^. However, these studies were uncontrolled, as the prevalence of AGA IgG positivity in a healthy control group was not assessed ^32, 37^. In addition, others have provided contradictory results ^30, 38^. Uhde *et al*. showed that NCGS is characterized by significant increased serum levels of soluble CD14, lipopolysaccharide-binding protein, and bacterial-directed antibodies (that is, flagellin), suggesting a systemic immune activation to microbial components ^39^. The same study, though not controlled against IBS, reported increased levels of a marker of epithelial integrity (that is, fatty acid-binding protein 2) which correlated with the markers of systemic immune activation, suggesting that mucosal barrier dysfunction may participate in NCGS ^39^. Notably, this last piece of evidence is in line with previous data showing epithelial barrier dysfunction in tissue explants obtained from gluten-related disorders ^40^. In contrast, no alteration in the lactulose/mannitol test was observed in patients with NCGS ^41^. Other mucosal abnormalities that would differentiate NCGS from other conditions such as IBS include a mild increase in intra-epithelial lymphocytes ^41, 42^, increased interferon-gamma gene expression ^42^, increased goblet cell number ^19^, and changes in Bacteroidetes-to-Firmicutes ratios ^19^.

## Conclusions

NCGS is an increasingly recognized clinical entity characterized by intestinal and extra-intestinal symptoms related to the ingestion of gluten-containing foods in patients in whom celiac disease and wheat allergy have been excluded. Clinically, NCGS is often indistinguishable from functional GI disorders and primarily from IBS. According to double-blind controlled studies, it appears that NCGS is overemphasized. The identification of reliable tests that could be used in clinical practice would enormously improve the recognition, legitimization, and treatment of this disorder.

## Abbreviations

AGA, anti-gliadin; ATI, amylase-trypsin inhibitor; DBPC, double-blind placebo-controlled; FODMAPs, fermentable oligosaccharides, disaccharides, and monosaccharides and polyols; GCD, gluten-containing diet; GFD, gluten-free diet; GI, gastrointestinal; HLA, human leukocyte antigen; IBS, irritable bowel syndrome; IBS-D, irritable bowel syndrome with diarrhea; NCGS, non-celiac gluten sensitivity; tTG, tissue transglutaminase
